# The Impact of Epicatechin on Human Cognition: The Role of Cerebral Blood Flow

**DOI:** 10.3390/nu10080986

**Published:** 2018-07-27

**Authors:** Crystal F. Haskell-Ramsay, Jeroen Schmitt, Lucas Actis-Goretta

**Affiliations:** 1 Department of Psychology, Northumbria University, Newcastle upon Tyne NE1 8ST, UK; 2 Nestlé Research Centre, Route du Jorat 94, 1000 Lausanne, Switzerland; Jeroen.Schmitt@rdls.nestle.com; 3 Nestlé Research Centre, 21 Biopolis Road, Singapore 138567, Singapore; Lucas.ActisGoretta@rdsg.nestle.com

**Keywords:** epicatechin, cocoa, cognition, cognitive, cerebral blood flow, mood, phenolic, polyphenol, phytochemical

## Abstract

Epicatechin is a monomeric flavanol found in food sources such as tea, apples, berries and cocoa. A number of large-scale epidemiological studies have demonstrated an association between the consumption of these foods and cognitive function, as well as improved blood flow. The aim of this review is to summarise the evidence from intervention studies to clarify the effect of epicatechin on cognition and to consider the role of increased cerebral blood flow as a mechanism for any effects. The effects of epicatechin as consumed in cocoa are, therefore, reviewed here as this represents the only dietary source where it is purported to be the major active component. Our main findings are that a) the positive modulation of tasks that involve memory, executive function and processing speed in older adults; b) the cognitive benefits are more often shown in studies containing more than 50 mg epicatechin/day; and c) all studies with a duration of 28 days or longer in populations >50 years old demonstrate a cognitive improvement. However, as highlighted by this review, it is not currently possible to attribute effects solely to epicatechin without consideration of synergies. In order to overcome this issue, further studies examining the cognitive effects of epicatechin in isolation are required. The role of cerebral blood flow also requires further investigation through simultaneous measurement alongside cognitive function.

## 1. Introduction

**Figure 1:**
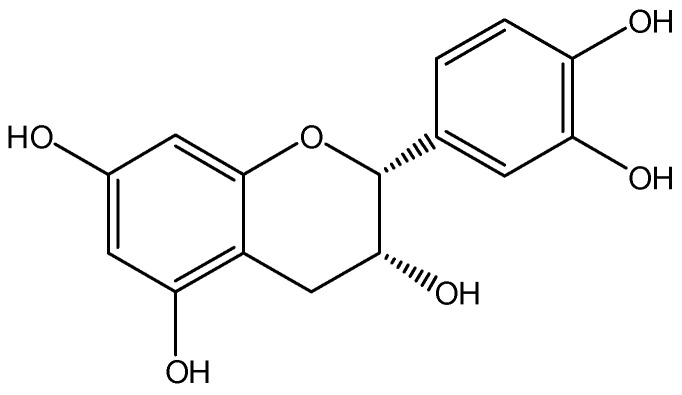
The chemical structure of (−)-epicatechin.

Flavanols are a subclass of the bioactive compounds, flavonoids, which can be further sub-divided into monomeric flavan-3-ols and their oligomeric/polymeric counterparts, procyanidins. Epicatechin (see Figure 1), along with catechin, is a monomeric flavan-3-ol, which is abundant in food sources such as tea, apples, berries, and particularly cocoa. A number of large-scale epidemiological studies have demonstrated a link between the consumption of these epicatechin-rich foods and cognitive function. For instance, the Paquid longitudinal study demonstrated that the relative risk of dementia was significantly lower for those in the two highest tertiles of flavonoid consumption, compared to those in the lowest tertile when followed up 5 years later [1]. In addition, analysis of neuropsychological function (Benton’s Visual Retention Test, Isaacs Set Test, Mini-Mental State Examination (MMSE)) at initial assessment of flavonoid intake (including flavanols), revealed a significant positive association between flavonoid consumption and task performance. An inverse relationship between intake and cognitive decline over the 10-year follow up was also demonstrated, whereby those in the highest two quartiles of flavonoid intake had less cognitive decline than those in the lowest quartile [2]. Other studies have provided data on specific sources of flavonoid. In a cross-sectional study of 1003 community-dwelling Japanese adults aged 70 years and above, Kuriyama et al. [3] demonstrated a significant inverse relationship between catechin-rich green tea consumption and cognitive impairment, as measured by MMSE, which was not observed for fermented black or semi-fermented oolong tea, or coffee. The role of flavonoids in this relationship is questioned by data from the Singapore Longitudinal Ageing Studies cohort showing a significant negative relationship between cognitive decline (MMSE) and black and oolong tea, but not green tea, in 2194 Chinese community-dwelling adults aged over 55 [4]. However, further analysis of a sub-sample of this cohort in a cross-sectional design employing a range of cognitive tasks showed that tea consumption was associated with the better performance across tasks, irrespective of whether fermented (black/oolong) or unfermented (green) [5]. Similarly, in the Norwegian Hordaland Health Study (HUSK), 2031 elderly (70–74 years) tea drinkers performed better in 4 out of 6 cognitive tests as compared to non-tea drinkers; wine drinkers also showed significantly better performance across all six tests versus non-wine drinkers, with this effect levelling off at 100 mL/day; and consumers of chocolate performed significantly better than non-consumers in 5 out of 6 cognitive tests, with a plateau in increased performance at 10 g per day [6].

Epidemiological studies suffer a number of well-documented problems, such as the difficulty in attributing cause and effect and uncontrolled confounds. However, an additional issue with the cohort data described above is that it does not allow conclusions regarding the role of epicatechin in these effects. In the case of wine consumption, findings are particularly difficult to interpret in relation to flavonoid content due to studies showing positive effects on cognition of moderate alcohol intake [7]. Similarly, as tea also contains caffeine and the amino acid l-theanine, it is difficult to determine the specific contribution of flavonoids to this relationship; and, in the case of green tea, the effects are often attributed to epigallocatechin-3-gallate (EGCG), rather than epicatechin. The limitations of epidemiological data require that in order to reliably assess the impact of epicatechin on the brain, it is necessary to consider data collected in randomised controlled trials. However, there are currently no published intervention trials examining the effects of isolated epicatechin on human cognition. Therefore, as cocoa represents the only dietary source where epicatechin is purported to be the major active component, this paper will review human randomised controlled trials exploring the role of epicatechin administered in cocoa on cognition. The potential mechanisms for the effects of epicatechin-rich cocoa on cognition will then be discussed with a particular focus on the role of cerebral blood flow.

## 2. Cognition Intervention Studies

In the first intervention study to assess the effects of epicatechin-rich cocoa on cognition, the impact of 5 days’ supplementation with 172 mg of cocoa flavanols (CF) (31 mg epicatechin) on letter pair switching performance was assessed. No effects were observed in healthy young females when compared to a matched control [8]. It is possible that the dose employed in this study was insufficient to produce behavioural effects, but another suggestion is that the healthy young participants were performing close to ceiling and benefits were therefore unlikely to be observed. This latter suggestion is supported by the demonstration of significant improvements to the performance of an intense 60-min Cognitive Demand Battery (CDB) in healthy young adults. Improvements to executive function and attenuation of mental fatigue increases induced by the demanding tasks were observed at 90 min post-administration of 520 and 994 mg of CF (94 and 184 mg epicatechin respectively) when compared to a control matched for macronutrient, mineral, caffeine and theobromine content [9]. Improvements to sustained [10] and selective attention [11] have been demonstrated in more recent studies following acute supplementation with CF. However, one study failed to find effects on a single 5-min Stroop task following 900 mg of CF [12], again potentially indicating issues with ceiling effects. The importance of task demands/fatigue is further highlighted by evidence showing that CF (447 mg epicatechin) can offset the negative effects of sleep deprivation on a working memory task at 90 min post-consumption in women [13]. Improvements to visual contrast sensitivity (as assessed by reading numbers that became progressively more similar in luminance to their background), time to detect motion direction, and visual-spatial memory were also shown in 18- to 25-year-olds, 90 min following acute consumption of dark chocolate as compared to white chocolate [14]. However, the use of a control that was not matched for methylxanthine or macro/micronutrient content makes it impossible to attribute the effects to phenolic compounds. This issue is overcome when comparing the effects of a cocoa tablet (3058 mg *Theobroma cacao* seed extract containing 250 mg CF) to cellulose in 18-to 40-year-olds [15]. Lower mental fatigue ratings prior to a shortened 30-min version of the CDB described above, as well as a greater number of serial sevens subtractions during the first repetition of the task were observed in the cocoa group when measured at 3 h post-tablet consumption. Stress ratings following 30 days’ supplementation were also higher following cocoa than placebo, an effect that is difficult to explain. It should be noted that each of the significant findings presented was the result of individual comparisons of the data at each time point. Therefore, given the isolated nature of these effects and the statistical approach adopted, it would be unwise to over-interpret these findings.

The finding of higher stress levels is also in contrast to findings from Pase et al. [16] showing increased calm and content ratings following CF supplementation. Cognitive and mood effects were assessed acutely and following 30 days’ supplementation in 40- to 65-year-olds using the standard 20-min Cognitive Drug Research (CDR) battery employed in numerous dietary intervention studies (e.g., [17,18]) and Bond-Lader mood scales [19]. Neither 250 nor 500 mg of CF (25 and 50 mg of epicatechin, respectively) impacted significantly on cognition or mood when measured acutely at 1, 2.5 and 4 h; however, increases in calm and content ratings were observed following 30 days’ supplementation with 500 mg of CF, which the authors suggest may be due to action on GABA_A_ receptors. Neurocognitive data from the same study showed no effects on a spatial working memory task assessed at baseline and following the 30-day supplementation. Steady State Visually Evoked Potentials (SSVEPs) recorded during task performance using Steady State probe Topography (SST), a form of electrophysiological brain imaging, revealed, perhaps surprisingly, that the pattern of posterior-parietal SSVEP amplitude in the 250 mg CF group, and to a lesser extent the 500 mg group, was significantly lower than the pattern observed following the control. Latency was also decreased in the same region following both the 250 and 500 mg CF interventions, indicative of increased neural processing speed [20]. In light of the lack of effects on cognition and the reduction in latency, it is suggested by the authors that this decrease in amplitude may reflect an increase in neural efficiency, whereby participants are able to perform at the same level with reduced activation. This assertion is supported by previous data showing a positive association between increased posterior-parietal activation and task difficulty [21]. It is interesting to note that this potential increase in neural efficiency seen following 250 mg is not accompanied by the modulation of mood demonstrated by the 500 mg dose, as presented in Pase et al. [16]. It has been suggested that a longer supplementation may be required to see robust improvements in cognition. Indeed, Brickman et al. [22] demonstrated that 12 weeks’ supplementation with 900 mg CF (138 mg epicatechin) led to participants in the high CF group responding 630 milliseconds faster than those consuming a matched low CF control on a Modified-Benton test, developed to localise the function to the dentate gyrus area of the hippocampus. This difference in reaction time equates to around three decades of ageing and was evinced as a slowing of responses from baseline to study end in the control group, as well as a faster response in the high CF group. The effects on reaction time were inversely related to cerebral blood volume changes in the dentate gyrus. These data are extremely exciting and provide an excellent basis for further study.

In addition to the longer intervention employed, Brickman et al. [22] also explored the effects in a slightly older cohort (50–69 years) and it may be that age played an important role in these effects. In the first study of the cognitive effects of cocoa in elderly adults, Crews et al. [23] failed to find any effects of 805 mg of CF (35 mg epicatechin) supplemented to over 60-year-olds (mean 68.7 years) for 6 weeks. However, the treatments were not matched for methylxanthine or micronutrient content and, importantly, the carbohydrate and energy levels were more than doubled in the control as a consequence of the use of sugar rather than the sweetener used in the cocoa condition. In addition, the low levels of epicatechin could explain the lack of cognitive effects. It is, therefore, difficult to interpret these null findings in relation to flavan-3-ols. Similarly, Sorond et al. [24] failed to find improvements to MMSE or Trail Making Tasks (TMT) A and B when measured at 24 h and 4 weeks following 1218 mg of CF (219 mg epicatechin) per day in older adults enrolled on the basis of hypertension or type II diabetes. However, significant improvements to global cognition and increases in brain-derived neurotrophic factor (BDNF) have been observed following 28 days supplementation with 494 mg of CF (89 mg epicatechin) in healthy elderly [25]. Furthermore, Desideri et al. [26] demonstrated improvements to cognition in elderly adults (65+ years) with Mild Cognitive Impairment (MCI) following 8 weeks’ supplementation. Compared to control, 520 and 994 mg of CF (95 and 185 mg epicatechin) led to faster completion times on TMT A and B and 994 mg also led to significantly improved verbal fluency performance compared to the control. Reduced insulin resistance (IR) as a consequence of CF consumption was found to explain ~40% of composite *z* score variability. In a replication of this work in healthy elderly adults, Mastroiacovo et al. [27] observed identical results to those shown in MCI with the exception that IR explained ~17% of composite *z* score variability. In both studies, there were no significant effects on MMSE, highlighting the lack of sensitivity of this measure to detect small changes in cognition over a short time period. It is currently unclear whether the lack of improvements reported by Sorond et al. [24], despite improvements on the same tasks in Desideri [26] and Mastroiacovo [27], relates to the shorter length of intervention, the population studied, or the high dose employed, and these factors all require further investigation. Similarly, the only study of acute effects in older adults failed to detect any improvement to cognition [28], a finding which may relate to the low dose of epicatechin (25 mg and 49 mg), small sample size or insensitive task selection. 

Seven out of nine studies that focused on the effects of a single dose of epicatechin-rich cocoa explored effects in young populations (<40 years). Of these, six showed positive effects upon cognition [9,10,11,13,14,15]. The only study to fail to show acute effects in a young population employed a small sample size (*n* = 12) and a single 2-part task lasting only 5 min [12]. Of the two studies in older adults (>40 years), one employed a small sample size [28] and the other explored effects in 40–65 years [16] who may be a particularly difficult age group to detect effects in due to the presence of undiagnosed underlying conditions with the potential to impact the findings. Of the 10 studies to explore the effects of repeated administration, 4 showed positive effects on cognition. Those studies that failed to find effects [8,15,16,20,23,24] tended to employ a shorter intervention period (5 days to 6 weeks), whilst positive effects were observed when administration continued for longer (8–12 weeks) [22,26,27]. One notable exception to this is the finding of a global improvement to cognition in older adults following 28 days’ supplementation [25]. This may be due to the combining of outcomes to increase the power or the use of a crossover design to minimise the impact of individual differences. Those studies showing a positive effect also employed a higher dose of epicatechin (>50 mg) than those failing to show effects. One exception to this is a lack of effects following 30 days’ supplementation with 219 mg of epicatechin [24], these null findings may relate to the length of intervention or to the inclusion criteria of hypertension and/or type II diabetes.

See Table 1 for a summary of randomised controlled trials assessing the impact of cocoa on cognition. 

## 3. Potential Mechanisms and Bioavailability

### 3.1. Potential Mechanisms

The exact mechanisms responsible for the effects of flavanols on brain health are yet to be determined but previous attributions to antioxidant properties have begun to be replaced by functions which impact synaptic plasticity via a cascade of cell signalling mechanisms, such as the modulation of receptor function, gene expression and protein synthesis, improved neuronal survival and increased spine density [29,30,31]. For instance, the treatment of mouse cortical cells with (−)-epicatechin in the range 30 nmol/L–30 µmol/L produced a bell-shaped dose-response on phosphorylation of cyclic adenosine monophosphate (cAMP) response element binding protein (CREB), with a maximum stimulation observed with doses between 100–300 nmol/L. This neuronal response was PI3-kinase and extracellular signal-regulated kinase (ERK) 1/2-dependent, and the phosphorylation of ERK 1/2 and Akt following (−)-epicatechin was shown to follow the same dose-response. Equivalent phosphorylation of ERK was also shown following the (−)-epicatechin metabolite, 3′-O-methyl-epicatechin, with no effects of epicatechin glucuronide. Eighteen hours after treatment with 100 nmol/L epicatechin, cAMP responsive element (CRE) mediated gene expression was shown to be up-regulated in a partially ERK-dependent manner, and the levels of GluR2 protein were increased [32]. Neuroprotective effects of (−)-epicatechin have also been demonstrated through decreased amyloid β-(Aβ) induced apoptosis [33], which is related, at least in part, to activation of c-Jun N-terminal protein kinase (JNK) and p38 mitogen-activated protein kinase (MAPK) [34,35,36].

### 3.2. Caveats for Result Interpretation

Difficulties in elucidating specific mechanisms of action for flavanols are due in part to the variety of compounds present when administered as food. For example, cocoa contains the methylxanthines caffeine and theobromine, along with fat, protein, carbohydrates and a range of minerals, all of which are usually matched in the control intervention but the total flavanol content includes monomers and procyanidins, which are not matched in the control, therefore presenting difficulties in ascribing effects solely to epicatechin. In addition, robust effects on biomarkers in vitro are often not replicated when measured in vivo. One explanation for this is that absorption is limited in vivo and extensive metabolism takes place in the small and large intestine, the liver and in cells. Therefore, due to extensive conjugation and metabolism, the substance administered may differ from that detected in systemic circulation [37,38]. Unlike dimer procyanidins, larger oligomeric flavanols present in cocoa were not detected in relevant levels in human plasma [39]. Previously, unmetabolised (−)-epicatechin has been detected in plasma at 2 h post-consumption of high flavanol cocoa drink (containing 917 mg (−)-epicatechin)) [40]. However, recent studies have failed to detect unmetabolised (−)-epicatechin compounds that were previously identified, suggesting inadequacies in the previous methodologies [41,42,43]. The identification and quantification of (−)-epicatechin conjugates was incomplete due to a lack of purified standards but new methods emerged indicating inadequacies in earlier methodologies. The rapid metabolism of epicatechin in the small intestine results in glucuronides, sulphates and/or methyl conjugates. For instance, following the consumption of 100 g of dark chocolate containing 79 mg of (−)-epicatechin, Actis-Goretta et al. [42] identified the following epicatechin metabolites as most relevant: epicatechin-3′-β-d-glucuronide (32%), epicatechin-3′-sulphate (24%), and epicatechin-3′-*O*-methyl-epicatechin sulphates substituted in the 4’, 5, and 7 positions. Despite the variable results, the evidence is emerging with regards the major metabolites and this is expected to continue as techniques and standards are developed. However, the studies described all suffer from extremely low sample sizes and given the large individual differences present it is essential that large-scale studies are conducted to explore the factors impacting on this. In particular, studies often differ with regards restrictions on diet in terms of polyphenol intake, ranging from no restrictions [40] to 12-h [44] and 48-h [42] restricted (‘beige’) diets. Differences also occur with regards length of fast from 2 h [44] to 12 h [40,42]. Given the importance of the mucosal integrity of the small intestine to absorption and the impact that small intestine microbiota has on this, it is important to explore the impact of dietary restrictions on bioavailability results. As the small intestine microbiota can fluctuate over days and even within a day [45], and as cocoa flavanols have shown prebiotic properties (albeit in faecal samples) [46], the possibility exists that restriction of polyphenol intake prior to bioavailability studies, negatively impacts on microbiota and this, in turn, reduces absorption from the small intestine. Therefore, those people who consume higher levels of polyphenols habitually may show increased absorption in intervention studies and, therefore, greater benefits over their counterparts with a lower level of intake. This also suggests that bioefficacy may be reduced in acute studies where a ‘beige’ diet is adhered to and this is something that warrants further investigation.

### 3.3. Blood-Brain Barrier

A further issue when considering the impact of epicatechin on brain health is its ability to reach the brain. Epicatechin has been shown to cross the blood-brain barrier (BBB) in vitro and also to be conjugated with glucuronic acid in these endothelial cells [47]. Data from animal studies have also shown epicatechin and its metabolites in brain tissue at pharmacologically relevant levels following oral administration [48,49] and despite data showing lower brain uptake of sulphated and glucuronidated derivatives in vitro [50], animal studies have shown that glucuronides can enter the brain [51,52]. However, the present knowledge on the ability of epicatechin and its metabolites to enter the brain is limited and in the absence of evidence for the biological activity of the metabolites, it is currently not possible to definitively conclude on the potential for epicatechin to exert direct effects on the brain. 

## 4. Peripheral and Cerebral Blood Flow Intervention Studies

As the potential for the direct effects of epicatechin on the brain has not been conclusively determined, indirect mechanisms have been explored. One such mechanism is improved blood flow. Several observational studies have linked habitual cocoa intake to lowered risk for a number of blood flow-related conditions such as high blood pressure, coronary heart disease, acute myocardial infarction, heart failure, carotid atherosclerotic plaques and strokes [53,54,55,56,57,58,59,60]. These data from epidemiological studies are supported by a number of intervention studies that have explored acute and chronic effects of cocoa on blood pressure [61,62,63,64,65,66,67,68] and platelet function [69,70,71,72,73]. Several studies have indicated a specific effect of cocoa on endothelial function as demonstrated by the increased flow-mediated dilation (FMD) whether studied chronically [62,64,68,74,75], acutely [65,75,76,77,78,79], or acutely superimposed upon a chronic increase [80,81]. Further support for the importance of endothelial function in this relationship comes from studies showing an absence of the modulation of endothelial-independent brachial artery diameter [64,76,80,81]. Mills et al. also demonstrated an improvement in FMD after the oral ingestion of epicatechin (200 mg) [82]. This effect on FMD was not replicated in older pre-hypertensive adults (40–80 years) when measured chronically or acute-on-chronically following 4-week supplementation with epicatechin (100 mg/day) [83]. Given the available data, it seems unlikely that the effects of epicatechin on FMD are only observed acutely but a dose of 100 mg/day was potentially not sufficient to see these effects in this population. Interestingly, the latter study employed epicatechin extracted from acacia heartwood with aqueous alcohol, possibly indicating an impact of the source and extraction method upon the quality of the epicatechin produced. This is potentially supported by data showing FMD increases in young and elderly participants alike both acutely and following 2-week epicatechin supplementation when administered in the form of cocoa [84].

In the first study to extend the findings of improved peripheral vascular function following cocoa consumption to cerebral blood flow (CBF), arterial spin labelling (ASL) magnetic resonance imaging (MRI) revealed significant increases in grey matter CBF 2 h post-consumption of 516 mg CF (93 mg epicatechin) as compared to control in healthy young adults [8]. This is supported by data from a recent study using the same technique showing regional increases particularly in the anterior cingulate cortex and the central opercular cortex of the parietal lobe in older adults (55–65 years) at rest [85]. The acute modulation of cerebral haemodynamics was also demonstrated by an increase in deoxygenated haemoglobin in younger adults measured using Near Infrared Spectroscopy both at rest and during the performance of a Stroop task [12]. Conversely, a reduction in CBF velocity measured with Transcranial Doppler (TCD) was reported following milk and dark chocolate, as compared to white chocolate, in post-menopausal women. As this reduction was observed during cognitive tasks with no effects on performance, the authors interpret this as indicating increased cerebrovascular efficiency [28]. Other studies employing TCD have failed to find effects on CBF velocity whether measured acutely (9) or following repeat administration [15,24,86] in samples ranging from 18-to 83-year-olds and employing a range of doses of epicatechin from 25–219 mg. The most salient explanation for these findings is the lack of sensitivity of TCD to detect small changes in cerebral blood velocity due to the variability in signal detection. Conversely, the blood oxygenation level dependent (BOLD) fMRI signal intensity was shown to be significantly increased following 5 days’ consumption of epicatechin-rich cocoa as compared to control. Given the previously demonstrated increase in grey matter CBF, it is not clear whether this increase in the BOLD signal indicates an increase in neuronal activity or merely reflects modulation of vascular function. However, as this modulation of activation was apparent in brain areas relevant to the task, it is perhaps surprising that no significant effects on cognitive performance were observed, a finding which indicates that this modulation is not always sufficient to produce measurable behavioural effects in healthy young adults. In the only study to date to demonstrate the modulation of cerebral perfusion in conjunction with cognitive change following CF, increases in cerebral blood volume (CBV) in the right hippocampal circuit were positively correlated with performance on a Modified Benton task in older adults [22].

See Table 2 for an overview of randomised controlled trials assessing the impact of cocoa on brain function.

## 5. Summary of Evidence for Cognitive Effects and Their Relationship to Blood Flow

Of the eight studies that have assessed the cognitive effects of cocoa in young adults, one showed positive effects that can be confidently attributed to flavan-3-ols [9]; four showed significant improvements but did not explicitly match for other active compounds such as methylxanthines and/or macronutrients [10,11,13,14]; others have shown no effects [8,12] or produced only a single effect [15]. Notably of the three studies to demonstrate robust effects, two employed cognitively demanding tasks [9,10] and the other showed effects following sleep deprivation [13]. This indicates that the likelihood of detecting improvements in young adults may be increased in states whereby ceiling effects are prevented. This may also relate to the proposed mechanism of action for such effects as cerebral blood flow is more likely to be compromised in times of increased cognitive demand and when sleep deprived. Neurovascular coupling ensures that increases in demand for the metabolic substrates glucose and oxygen, as a consequence of neuronal activity, are met by increased cerebral blood flow. However, tasks or paradigms that are particularly difficult or fatiguing may increase demands to a level whereby it is possible that the increased supply of substrates may be beneficial. Similarly, CBF has been shown to be reduced both in sleepwalkers when compared to controls [87] and following 48-h acute deprivation [88], particularly in the prefrontal cortex. Therefore, as an impairment to neurovascular coupling is rare in healthy young adults, increases in CBF may only result in cognitive improvements when this is compromised either through increased demand or reductions in supply, for example following sleep deprivation.

A lack of cognitive effects has been shown in middle-age [16,20], which may reflect the complexities inherent in assessing cognition in those potentially suffering currently undiagnosed comorbidities. However, studies in older adults have shown positive effects on cognition in four out of seven studies. Improvements were shown to trail making and verbal fluency [26,27] and to object recognition [22], as well as global cognition [25]. Importantly, the only one of these studies to measure cerebral blood flow showed a significant correlation between change in cognition and change in CBV [22]. Of the three studies in older adults that showed null findings on cognition, the first is likely due to mismatches in sugar and sweetener levels between the placebo and active treatments [23]. The second [24] potentially relates to the 4-week intervention employed and the diagnosis of participants as hypertensive or type II diabetic. Although significant improvements have been reported in older healthy participants following 4 weeks’ CF consumption [25], it is possible that a longer supplementation is required in those diagnosed with clinical impairment to vascular function. No significant effects were observed in healthy elderly following acute supplementation [28], which potentially supports the assertion that an increased length of administration may be required in accordance with vascular function senescence. However, as cerebral vascular function decreases with age, as evidenced by reductions in cerebral blood velocity [89], activation, and coupling between oxygenated and deoxygenated haemoglobin [90], it is possible that effects of epicatechin are more likely to be detected in older populations. The reasons for this are two-fold, as vascular risk factors are associated with cognitive impairment [91], this decline with ageing helps to eradicate the confound of ceiling effects suggested in healthy young adults; and declines in cerebrovascular function with age provide a mechanism for impacting cognition through improvements to vascular health. This latter suggestion is supported by data showing that CF induced improvement in endothelial function was greatest in older adults relative to younger adults [92].

Endothelial function decreases with age as the capacity to produce nitric oxide (NO) diminishes. NO formed by endothelial NO synthase (eNOS) has a number of impacts on vascular health such as increased vasodilation and blood flow and decreased vascular resistance, hypotension, platelet aggregation and adhesion [93]. Increases in plasma nitric oxide metabolites have been observed within one hour of consumption of flavanol-rich food [40,77] and following (−)-epicatechin in isolation [94] and a wealth of evidence demonstrates the importance of this to effects of CF on peripheral blood flow effects. Acutely, plasma nitric oxide species have been positively correlated with FMD [76] and a reversal of this was observed following the inhibition of nitric oxide synthase by intravenous infusion of L-NG-monomethyl-arginine nitrite (LNMMA) [77]. Chronically, increases in nitrite (an oxidative metabolite of nitric oxide) [80] have been observed, as well as circulating angiogenic cells [68], which are linked to endothelial nitric oxide synthase [95]. The effects of NO are particularly important with regards cerebrovascular function due to the tight control of this system and the reliance on a continuous flow of blood to supply neural substrates. Therefore, in addition to effects on basal CBF of endothelial NO, neuronal NO plays a crucial role in regulating neurovascular coupling to ensure neuronal activity is met by sufficient increases in CBF [96], as well as impacting neuroplasticity [97]. The role of NO in CBF effects of ageing is highlighted by the observation that L-arginine (the immediate precursor to NO) increased the cerebral blood flow velocity to a lesser extent in older adults (~70 years) than younger ones (~29 years) [98], and that a decrease in CBF is demonstrated in older but not young adults following infusion of L-NMMA [99], suggesting that the NO pathway is less involved in the regulation of basal blood flow in young subjects, or that mechanisms exist to compensate for its disruption. This latter finding supports the suggestion that cognitive improvements via increased CBF may be less likely to be observed in healthy young adults. Therefore, in order to further understand the relationship of CBF to the cognitive effects of epicatechin and to further elucidate the role of NO in any effects, it is essential that future studies concurrently measure CBF and cognition. It is particularly important that CBF is measured at rest and during task performance in order to disentangle any global effects on CBF from those relating to neuronal demand. It is also worth noting that whilst the focus of this review is the role of CBF, the inclusion of other brain MRI measures such as neuroinflammation, white matter disease, and amyloid deposition/clearance would help elucidate the impact of epicatechin on cognition.

## 6. Conclusions and Future Directions

At present, it is difficult to make definitive conclusions regarding the impact of the consumption of epicatechin on cognition due to a paucity of data. However, evidence is emerging which suggests a positive modulation of tasks that involve memory, executive function and processing speed in older adults following cocoa flavanol (CF) interventions where the major active component is purported to be (−)-epicatechin [22,25,26,27]. Due to a lack of consistency in tasks employed across studies, direct replication of these effects cannot be confirmed or disputed in young adults, but similarities exist whereby improvement has been shown to tasks which involve varying contributions of information processing, working memory, and psychomotor speed following CF [9,10,13,15]. In order to further explore the role of age in any effects, it is necessary to design studies that include older and young adults, particularly in light of age-associated reductions in eNOS expression [100]. In addition, as cognitive effects have tended to only be measured acutely in young adults whereas effects in older adults have typically involved ≥4 weeks’ intervention, the impact of the length of intervention is also something that requires further clarification in relation to age. This is particularly apparent when considering that improvements to calm and content mood ratings were shown following 4 weeks’ intervention in middle-aged adults, but not acutely [16]. Moreover, the length of the intervention may represent an important difference between observational and intervention studies given the longest supplementation period to date is 12 weeks. It is therefore important to conduct longer studies which also include a follow-up period to assess the longevity of effects. This would add to data from studies of phenolic-rich grape juice showing carryover effects indicative of enduring benefits whether consumed for 12 weeks [101] or acutely [102]. Furthermore, differences in response to epicatechin as a function of sex have generally not been explored, as is often the case in nutrition intervention studies, and this is something that should be addressed, particularly in light of cognitive effects shown in females only following sleep deprivation [13]. 

In terms of dose, studies to date have employed doses of CF ranging from 172–994 mg with reported epicatechin levels ranging from 25–447 mg. With the exception of studies in middle-aged participants, studies including those selected on the basis of a clinical diagnosis, and one study that may have been underpowered, positive cognitive effects were shown in studies employing doses ≥494 mg CF/day. These studies provided supplements that contained more than 50 mg epicatechin/day were reported. However, the level of epicatechin is not always reported and interventions differ in other aspects of composition making it difficult to conclusively attribute effects to epicatechin level. This highlights the need for further studies which delineate the role of epicatechin, either through the design of studies that allow effects of epicatechin-rich foods to be attributed to epicatechin or through the supplementation of epicatechin, and its metabolites where possible, in isolation. A further method to elucidate the role of epicatechin, and particularly metabolites, is to include the blood measures of these compounds and examine the relationship between these and cognitive outcome measures. 

Mechanistically, although NO has been highlighted as an important pathway for blood flow effects of CF and improvement to endothelial function has been suggested as a mediator in cognitive effects of CF, this relationship is extremely complex and requires further exploration along with other potential mechanisms of action. In older adults, increases in CBV have been correlated with cognitive improvements but this finding requires replication and other factors should be considered alongside blood flow effects. In the case of younger adults, although blood flow to the brain has been shown to increase following CF, this mechanism has not proved sufficient to produce cognitive improvements. This does not rule out this mechanism of action in certain circumstances but does provide evidence that increases in CBF do not necessarily lead to cognitive benefits. In order to further clarify the role of NO in modulations to CBF and cognition, methodologies similar to those in FMD studies should be employed whereby plasma nitric oxide metabolites were measured [40,77,94] and the impact of inhibition of nitric oxide synthase on blood flow effects was assessed [77,103]. Interestingly, 100 mg/day of (−)-epicatechin failed to increase FMD or NO in pre-hypertensives despite significant decreases in the related parameters of fasting insulin and insulin resistance [83]. Given its association with endothelial function and blood flow, the role of insulin and insulin resistance requires further exploration in relation to epicatechin, particularly as it has been shown to predict the overall cognitive performance changes following CF supplementation [26,27]. Finally, future studies of epicatechin should also consider the effects on mood and the potential for this to modulate cognitive effects. Pase et al. [16] demonstrated a significant improvement to content and calm ratings following 30 days’ CF supplementation, which they suggest may relate to effects on GABA_A_ receptors. However, mental stress also impairs endothelial function [104], so it is possible that demand is necessary to impair this in order to see effects in young subjects; this should be explored by employing tasks that allow modulation of demand in order to investigate the impact of this across ages, whilst concurrently measuring mood. 

Although promising evidence is beginning to emerge with regards the impact of (−)-epicatechin on cognition, it is not currently possible to attribute effects solely to epicatechin without consideration of synergies. In order to overcome this issue, future studies require the use of epicatechin in isolation. In addition, to avoid the issue of null findings on cognition often observed in nutritional intervention studies, it is essential that appropriate, hypothesis-driven tasks, are employed. Similarly, although studies including 28 days or more supplementation in >50-year-olds all showed positive effects on cognition, there is a lack of evidence regarding the optimal dose and time-frame for cognitive effects. Therefore, large-scale studies are needed that take these factors into account along with age, sex and habitual diet, whilst also assessing bioavailability and correlating this with any cognitive effects. 

## Figures and Tables

**Table 1:** Randomised controlled trials assessing the effects of cocoa on cognition.

Reference	Sample	Design	Dose, Duration	Methods	Effects of Epicatechin
Francis et al. (2006) [8]	*n* = 16 females (18–30 years)	RDBPC crossover	172 mg CF (31 mg epicatechin). Five days	Switch task; fMRI; HR (1.5 h PD)	Increased BOLD response in the dorsolateral prefrontal cortex, parietal cortex and ACC. No effects on cognition.
Scholey et al. (2010) [9]	*n* = 30 (18–35 years; mean 22)	RDBPC crossover	520 mg CF; 994 mg CF (94/184 mg epicatechin). Acute	2 serial subtraction tasks (3 and 7 s); RVIP; mental fatigue; STAI-Y1 (1.5 h PD)	Increased correct serial 3 subtractions; 94 mg attenuated mental fatigue; 184 mg improved RVIP RT but increased serial 7 subtraction errors.
Boolani et al. (2017) [10]	*n* = 23 (17 male) (mean 20 years)	RDBPC crossover	499 mg CF (epicatechin NK); 499 mg CF+70 mg caff; 66 mg caff. Acute	Serial subtractions (3 and 7 s); Bakan; CPT; motivation; mood; salivary methylxanthines (baseline and 22, 60 and 98 min PD)	CF vs placebo: decreased Bakan RT and FA. CF vs. CF + caff: decreased Bakan correct and increased omission errors. CF + caff vs. caff: decreased anxiety.
Tsukamoto et al. (2018) [11]	*n* = 10 males (mean 23 years)	RSBPC crossover	563 mg CF (epicatechin NK)	2-part Stroop; Face-name matching; FAS; mental fatigue; concentration; motivation: HR; MAP; glucose; lactate (baseline and 30 and 60 min PD at rest and 100, 130 and 160 min PD after exercise)	Improved Stroop interference.
Decroix et al. (2016) [12]	*n* = 12 male (mean 30 years)	RDBPC crossover	900 mg CF (185 mg epicatechin). Acute	2-part Stroop; NIRS; BDNF (baseline and 95 min PD at rest and 145 min PD after exercise)	Increased ΔHbO_2_ during word-colour Stroop at rest.
Grassi et al. (2016) [13]	*n* = 32 (16 male) (mean 25 years)	RDBPC crossover	520 mg CF (447 mg epicatechin). Acute	KSS; PVT; 2-back; FMD; BP; PWV (baseline ‘sleep’ condition and 90 min PD in ‘deprivation’ condition following one-night total sleep deprivation)	Preserved 2-back accuracy in women after ‘deprivation’. SBP, DBP and pulse pressure lower after CF vs. control. Negative effects of ‘deprivation’ on FMD and PWV counteracted by CF. FMD correlated with 2-back accuracy in ‘sleep’.
Field et al. (2011) [14]	*n* = 30 (18–25 years)	RSBPC crossover	773 mg CF (epicatechin NK). Acute	CS; motion coherence threshold; motion integration time threshold; visual SWM; CRT (2 h PD)	Improved CS, improved WM accuracy and speeded motion integration and CRT.
Massee et al. (2015) [15]	*n* = 40 (18–40 years, mean 24)	RDBPC parallel groups	250 mg CF (epicatechin NK). Acute/30 days	TCD of CCA, SUCCAB, CDB × 3, mental fatigue and stress before and after CDB, (baseline and 2 h acutely and at 30 days)	Decrease in fatigue pre-CDB and increase in sevens correct during the first repetition acutely. Stress lower at 30 days in the placebo group.
Pase et al. (2013) [16]	*n* = 72 (40–65 years)	RDBPC parallel groups	250 mg CF; 500 mg (25/50 mg epicatechin). Acute/30 days	Immediate WR; Simple RT; DV; Choice RT; Tracking; Spatial WM; Numeric WM; Delayed WR; Word Recognition; Picture Recognition; Bond-Lader VAS (baseline, 1, 2.5 and 4 h acutely and at 30 days)	Increased calm and content at 30 days.
Camfield et al. (2011) [20]	*n* = 63 (40–65 years; mean 52)	RDBPC parallel groups	250 mg CF; 500 mg CF (25/50 mg epicatechin). Thirty days	SST-SSVEP; SWM (baseline and PD)	Decreased SSVEP amplitude (25 mg) and increased latency (25 and 50 mg) in posterior parietal regions.
Brickman et al. (2014) [22]	*n* = 37 (50–69 years)	RDBPC parallel groups	900 mg CF (138 mg epicatechin). Twelve weeks	ModBent; CBV dentate gyrus (fMRI) (baseline and PD)	ModBent RT 630 ms faster in CF vs. control. CBV enhanced in CF; a correlation between change in cognition and CBV.
Crews et al. (2008) [23]	*n* = 90 (≥60 years; mean 69) MCI (≥24 MMSE)	RDBPC parallel groups	755 mg CF (epicatechin NK). Six weeks	BSRT; WMS-III Faces I and II; TMT; Stroop; WAIS-III DSST; total cholesterol (HDL, LDL, VLDL); triacylglycerol; C-reactive protein (baseline and 6 weeks). A-DACL General Activation; SBP; DBP; HR (baseline, and at 2 h PD at 3 and 6 weeks)	No positive effects. CF increased HR (3 and 6 weeks).
Sorond et al. (2013) [24]	*n* = 60 (mean 73 years) hypertension and/or T2D	RDBPC parallel groups	1218 mg CF (219 mg epicatechin). A duration of 24 h/30 days	MMSE; TMT-A and B; TCD of MCA to assess neurovascular coupling (baseline and PD)	No effects in the primary analysis.
Neshatdoust et al. (2016) [25]	*n* = 40 (22 male) (62–75 years, mean 68)	RDBPC crossover	494 mg CF (89 mg epicatechin). Twenty-eight days	Go-No-Go; Stroop; plus-minus; TMT; letter memory; free and delayed WR; word and face recognition; serial sevens; spatial delayed recall; virtual 3D radial arm maze; word stem completion; DSST; RVIP (baseline and PD)	Significant increase in global cognition and BDNF.
Desideri et al. (2012) [26]	*n* = 90 (65–82 years) MCI	RDBPC parallel groups	520 mg CF; 993 mg CF (95/185 mg epicatechin). Eight weeks	MMSE; TMT-A and B; VF (baseline and PD)	Increased speed of TMT-A and TMT-B. A total of 185 mg improved VF. Reduced IR, BP and LP, with IR explaining ~40% of composite *z* score variability
Mastroiacovo et al. (2015) [26]	*n* = 90 (~69 years)	RDBPC parallel groups	520 mg CF; 993 mg CF (95/185 mg epicatechin). Eight weeks	MMSE; TMT-A; TMT-B; VF (baseline and PD)	Increased speed of TMT-A and TMT-B. A total of 185 mg improved VF. Reduced IR, BP and LP, with IR explaining ~17% of composite *z* score variability.
Marsh et al. (2017) [28]	*n* = 12 (post-menopausal women) (77 years)	RSBPC crossover	200 mg CF; 395 mg CF (25 mg/49 mg epicatechin). Acute	Detection task; *n*-back (1 and 2 back); list learning and recall; continuous paired-association learning (separate day baseline and 60 min PD). FMD; MAP; HR; TCD of MCA (baseline and 60 min PD).	Milk and dark chocolate increased FMD, decreased CBV at rest and during tasks with no effects on cognition or MAP.

ACC = Anterior Cingulate Cortex; A-DACL = Activation-Deactivation Adjective Check List; BDNF = Brain-Derived Neurotrophic Factor; BOLD = Blood Oxygenation Level-Dependent response; BSRT = Buschke Selective Reminding Test; Caff = Caffeine; CBV = Cerebral Blood Velocity; CF = Cocoa Flavanols; CS = Contrast Sensitivity; DBP = Diastolic Blood Pressure; DSST = Digit Symbol Substitution Task; DV = Digit Vigilance; FMD = Flow-Mediated Dilatation; fMRI = functional Magnetic Resonance Imaging; HbO_2_ = Deoxygenated haemoglobin; HDL = High-Density Lipoprotein; HR = Heart Rate; IR = Insulin Resistance; LDL = Low-Density Lipoprotein; LP = Lipid Peroxidation; MAP = Mean Arterial Pressure; MCA = Middle Cerebral Artery; ModBent = Modified-Benton test; MMSE = Mini Mental State Examination; MRI = Magnetic Resonance Imaging; NK = Not Known; PD = post-dose; PWV = Pulse Wave Velocity; RT = Reaction Time; RVIP = Rapid Visual Information Processing; SBP = Systolic Blood Pressure; SST = Steady State Topography; SSVEP = Steady State Visually Evoked Potential; STAI-Y1 = State Trait Anxiety Inventory state component; TCD = TransCranial Doppler; TMT = Trail Making Test; VAS = Visual Analogue Scales; VF = Verbal Fluency; VLDL = Very Low-Density Lipoprotein; WAIS-III = Wechsler Adult Intelligence Scale-III; WM = Working Memory; WMS-III = Wechsler Memory Scale-III; WR = Word Recall.

**Table 2:** Randomised controlled trials assessing the effects of cocoa on brain function.

	Age	18–40										40–65				>50				References
Duration																				
Acute		[8]	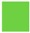	[9,10]	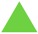	[11,13,14]	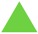	[12]	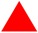	[15]	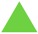	[16]	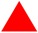			[28]	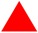	[85]	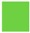	Francis et al. (2006) [8]; Scholey et al. (2010) [9]; Boolani et al. (2017) [10]; Tsukamoto et al. (2018) [11]; Decroix et al. (2016) [12]; Grassi et al. (2016) [13]; Field et al. (2011) [14]; Massee et al. (2015) [15]; Pase et al. (2013) [16]; Marsh et al. (2017) [28]; Lamport et al. (2015) [85]
					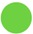				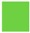		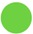		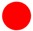				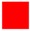			
											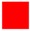									
1–14 days		[8]	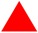													[24]	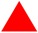	[86]	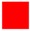	Francis et al. (2006) [8]; Sorond et al. (2013) [24]; Sorond et al. (2008) [86]
			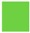														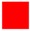			
28–30 days		[15]	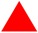									[16]	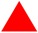	[20]	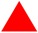	[25]	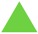			Massee et al. (2015) [15]; Pase et al. (2013) [16]; Camfield et al. (2011) [20]; Neshatdoust et al. (2016) [25]
			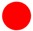										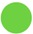		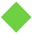					
			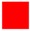																	
6 weeks																[23]	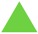			Crews et al. (2008) [23]
8 weeks																[26]	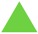	[27]	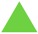	Desideri et al. (2012) * [26]; Mastroiacovo et al. (2015) [27]
12 weeks																[22]	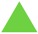			Brickman et al. (2014) [22]
																	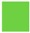			

Triangle = cognition; circle = mood; square = cerebral blood flow; diamond = steady-state visually evoked potential. Green = positive effect; red = no or negative effect. * = Mild cognitive impairment.
